# Applying the effort-reward imbalance model to household and family work: a population-based study of German mothers

**DOI:** 10.1186/1471-2458-12-12

**Published:** 2012-01-06

**Authors:** Stefanie Sperlich, Richard Peter, Siegfried Geyer

**Affiliations:** 1 Medical Sociology, Hannover Medical School, Carl-Neuberg Str. 1, 30625 Hannover, Germany; 2 Institute of Epidemiology, Ulm, Helmholtzstr. 22, 89081 Ulm, Germany

**Keywords:** effort-reward imbalance, household and family work, women, health

## Abstract

**Background:**

This paper reports on results of a newly developed questionnaire for the assessment of effort-reward imbalance (ERI) in unpaid household and family work. Methods: Using a cross-sectional population-based survey of German mothers (n = 3129) the dimensional structure of the theoretical ERI model was validated by means of Confirmatory Factor Analysis (CFA). Analyses of Variance were computed to examine relationships between ERI and social factors and health outcomes.

**Results:**

CFA revealed good psychometric properties indicating that the subscale 'effort' is based on one latent factor and the subscale 'reward' is composed of four dimensions: 'intrinsic value of family and household work', 'societal esteem', 'recognition from the partner', and 'affection from the child(ren)'. About 19.3% of mothers perceived lack of reciprocity and 23.8% showed high rates of overcommitment in terms of inability to withdraw from household and family obligations. Socially disadvantaged mothers were at higher risk of ERI, in particular with respect to the perception of low societal esteem. Gender inequality in the division of household and family work and work-family conflict accounted most for ERI in household and family work. Analogous to ERI in paid work we could demonstrate that ERI affects self-rated health, somatic complaints, mental health and, to some extent, hypertension.

**Conclusions:**

The newly developed questionnaire demonstrates satisfied validity and promising results for extending the ERI model to household and family work.

## Background

The health-related impact of qualitative psychosocial work characteristics has been amply demonstrated, but research has predominantly focused on paid labour. In this context, a variety of studies have examined the health-related impact of effort-reward imbalance (ERI) [1]. This model postulates that work characterized by high effort and low reward reflects a state of failed reciprocity between 'costs' and 'gains'. In case of an imbalance between high effort and low reward strong emotions of anger and frustration may emerge resulting in a sense of being treated unfairly. In the long run sustained activation of the autonomic nervous system may contribute to the development of physical and mental disease [2,3]. A specific personality trait characterized by excessive work-related overcommitment increase the risk of ERI and is the most important reason for maintaining a high cost/low gain condition. Although the ERI model has primarily been used as a theoretical framework for explaining work-related health risks, Siegrist [4] stresses that reciprocity of exchange is not confined to working life. It may be experienced in a similar way also in other domains, such as marital and parental relationships. Against this background, Knesebeck and Siegrist [5] extended the ERI model to social relationships beyond paid work, namely marital and parental roles and less specific civic roles. Li et al. [6] applied the model to school settings in order to analyse students' psychosocial stress and its association with self-rated health in adolescents. These studies provided first evidence that the ERI model may also be important outside the work-life.

As far as we know no studies exist where Siegrist's model had been applied to unpaid household and family work. However, there are analogies between paid and unpaid work justifying such an extension. Firstly, similarly to paid employment, household and family work are defining one's social identity and social status to a substantial degree. Secondly, unpaid work might be equally demanding, and some services such as caring, cooking and cleaning can also be purchased on the labour market. Thirdly and lastly, household and family work may also offer 'rewards' in terms of promoting self-esteem and may therefore provide the potential of experiencing a favourable self-concept.

However, there are also important differences between paid and unpaid work need to be taken into account. Constraints of demands in household and family work are often less pervasive and less obvious. Rewards received are more often of an emotional than of a material nature [5]. For that reason, rewards as assessed with the original ERI questionnaire in terms of 'financial and career-related rewards', 'esteem rewards' and 'gratification of job security' cannot be generalized to unpaid work. Thus, we developed a new questionnaire taking the specific efforts given and rewards received in household and family work into account. Initially, a review of the literature was carried out to identify the relevant aspects of effort and reward in this domain. This review revealed that some crucial efforts of the original ERI also hold for work at home, in particular 'time pressure', 'interruptions and disturbances' and 'pressure to work overtime'. A more specific expression of effort in household and family work was 'unappreciated work' and 'the feeling of being available to others'. The household and family work was characterised as 'diffuse' and 'never-ending' [7-9]. With respect to 'rewards' the review yielded that this work foremost provides interpersonal rewards, particularly with respect to child care [8]. Baruch and Barnett [10] concluded that women found the most rewarding aspects of the mother-role in the love of children, liking the kind of people they are, and pleasure in their accomplishments. For the role as a wife they also identified aspects related to the partner as important, in particular having an emotionally supporting partnership. Another source of reward is the meaning of the role as a mother and wife in persons' self-conception. In this perception a high value of being a mother and taking care for the family provides self-rewards resulting in better psychological well-being [11-13]. On the other hand, it was reported that household and family work has little institutional recognition and social prestige, and the recognition of being a housewife has significantly decreased over the last decades [8].

On the base of the literature review questionnaire items were generated and discussed with mothers as part of qualitative interviews. With their feedback in mind the questionnaire was developed and tested for reliability using clinical data of mothers with burnout [14].

This paper reports findings of this questionnaire using data of a cross-sectional population survey of German mothers. In more detail, the study addresses the following questions:

1.) Can the theoretical model of ERI in household and family work be confirmed in a population sample?

2.) Does ERI differ with respect to socio-demographic factors and family-related characteristics?

3.) Is lack of reciprocity in household and family work associated with increased health risks in mothers?

## Methods

### Sample

The sample consists of 3129 mothers living in Germany with minor children. The cross-sectional survey was conducted by the Institute TNS Healthcare on behalf of the Department of Medical Sociology at Hannover Medical School. Ethical approval was not required for this study. The data were collected in 2009 by means of a mail survey. The sample was derived from the Healthcare Access Panel comprising 75.000 households in total and 27.038 households with women having minor children. The Healthcare Access Panel is composed of respondents who have given their general consent to participate in surveys. Based upon an estimated response rate of 50 percent and a targeted case number of 2.500 mothers a total of 5.000 mothers were selected randomly out of the panel. The gross sample was drawn according to predefined quotas, i.e. age of mother and youngest child, school education, family status and number of children. The initial case number of young mothers (≤ 25 years) had to be completed by another 107 cases in order to meet the quota. Of these 5107 mothers 3183 have participated in the survey, corresponding to a return rate of 62.3%. A total of 54 mothers were excluded subsequently due to failing inclusion criteria (in particular youngest child was > 18 years of age). The sample was weighted according to German federal states, school education, mother's age, family status and number of children and thus can be considered as representative for German mothers regarding these characteristics.

### Measurements

#### Effort-reward imbalance (ERI) in household and family work

For measuring ERI in household and family work the newly developed questionnaire was used. In the instruction it was stated that the phrase "household and family work" includes a wide range of activities, including family organization, child care, help with homework, providing transportation for the children, as well as cooking, washing, tidying up, shopping, cleaning and much more. The component 'effort' was measured by eight items referring to demanding aspects of work environments of mothers by emphasizing quantitative workload. Response formats were constructed analogous to the originally ERI. First, subjects may agree or disagree whether the item content describes a typical feature of their work situation. Subsequently, mothers who agree are asked to rate to what extent they usually feel distressed by this experience. Every item has five categories ranging from (1) 'Yes, but this does not burden me at all' to (5) 'Yes, and this burdens me very greatly'. A sum score of these ratings was constructed as the unidimensionality of the scale had been documented by factor analysis analogous to the original ERI questionnaire [15]. Thus, a total sum score based on the eight items measuring effort varies between 8 and 40. The higher the score, the higher the demands. The component *Reward *is measured by 11 items, divided into four subscales: (1) intrinsic value of family and household work (3 items), (2) societal esteem (3 Items), (3) recognition from the spouse/partner (3 items), and (4) affection from the child(ren) (2 items) (see additional file 1: questionnaires). The answering and scoring procedures were the same as for the effort items. A score of 11 indicates the perception of the lowest distress due to lack of reward whereas a score of 55 reflects a very high distress.

Analogous to Siegrist et al. [15] the effort-reward ratio was computed for each respondent according to the formula: e/(r × c) where e is the sum score of the effort scale, r is the sum score of the reward scale (with reversed polarity, that means low scores indicate high distress due to lack of recognition) and c defines a correction factor for different numbers of items in the nominator and denominator. The correction factor is 0.73 if the nominator contains eight items (8/11). As a result, a value close to zero indicates a favourable condition (relatively low effort, relatively high reward), whereas values above 1.0 indicate an effort-reward imbalance, e.g. a high amount of effort spent that is not met by the rewards received in turn. As a predictor of health outcomes this ratio was transformed into a binary variable (values ≤ 1 vs. > 1). In order to differentiate mothers with slight, moderate and marked imbalance, mothers with a ratio > 1 were divided into three equally sized groups, namely: 1. ratio score ≤ 33^th ^percentile (slight imblance), 2. ratio score ranging from the 34^th ^to 65^th ^percentile (moderate imbalance), and 3. ratio score reached values above the 65^th ^percentile (marked imbalance).

##### Overcommitment

For assessing the personal component of the model the short version of the overcommitment questionnaire by Peter et al. [16] was transferred to household and family work including the dimension 'inability to withdraw from work obligations'. Only moderate linguistic changes were necessary, but two items had to be excluded because of small correlations with the overall overcommitment score. Each item has four categories ranging from (1) 'totally disagree' to (4) 'totally agree'. A sum score based on the four items varies between 4 and 16. The higher the score, the higher overcommitment as personality trait is pronounced. Women who answered on average at least each item with 'agree somewhat' (sumscore ≥ 12) were attributed to have excessive work-related commitment ('overcommitment').

The questionnaires of effort-reward imbalance and overcommitment were translated from German into English and independently back-translated to German by a professional translation agency (questionnaires see appendix).

#### Social and family-related characteristics

*Socioeconomic status *was measured using the following variables: school education, employment status and per capita income. Per capita income was calculated as follows: A weighting of '1' was assigned to heads of household. Each further adult got a weighting of 0.7 and every child a weighting of 0.4. *Family characteristics *were assessed by the following variables: single motherhood (i.e. living alone with at least one dependent child in the household), number of children in the household, age of youngest child, the women's perception of the division of housework (question: "who is responsible for housework, i.e. for all work arising such as cleaning, washing, caring and cooking?") and negative work-family spillover. Negative work-family spillover describes the issue that paid work interfered with functioning at home. The women's perceived extent of spillover was measured with the following three statements [17]: 1. "Due to my employment I am often too tired for joint activities with my partner/my child/ren", 2. "Due to my employment I am often so exhausted that household and family work are setting me under strain" and 3. "My partner/my family is annoyed that I am absorbed by occupational affairs when being at home". Each statement has five categories ranging from (0) 'no, not applicable to (4) 'yes, and this burdens me very greatly'. A new variable was calculated for analysis with three categories: 1 = no spillover (each of the items was answered with 'not applicable' or 'applicable, but does not burden me'), 2 = moderate spillover (at least one item was answered with 'burdens me somewhat') and 3 = marked spillover (at least one item was answered with 'burdens me grately' or 'very greatly').

#### Health outcomes

*Anxiety *and *depression *were assessed using the Hospital Anxiety and Depression Scale German Version (HADS-D) [18]. Each subscale contains seven items with four categories ranging from 0 to 3. Consequently, each subscale is ranging from 0 to 21.

A modified version of von Zerssen's complaints scale [19] was used for assessing *physical disabilities and discomfort*. Each item has four categories ranging from 'not at all' (0) to 'strongly' (3). In order to prevent confounding with the HADS-D all complaints with correlations with HADS-D subscales above r = 0.50 were not used for computing the sum score. This was the case with 6 of the 24 items of the complaint scale. Consequently, the sum score based on 18 items varying between 0 and 54.

*Subjective health status *measures respondent's evaluations of their satisfaction with health and has a range of five categories from 1 ('very poor') to 5 ('very good'). For measuring *hypertension *the mothers were asked if a doctor had ever diagnosed high blood pressure (response format 'yes' or 'no').

### Analyses

Confirmatory factor analysis was performed for testing the factorial structure of the theoretical model using the statistical package AMOS 6. Goodness of fit was assessed by the (Adjusted) Goodness of Fit Index, (AGFI, GFI) as well as Chi-square and Root Mean Square Error of Approximation (RMSEA). AGFI and GFI indicate the amount of variance and covariance explained by the model. RMR is the square root of the mean of squared discrepancies between the implied and the observed covariance matrices. Due to the fact that data were not normally distributed the asymptotical distribution-free method was applied for estimating parameters. We computed single factor variance analyses (ANOVA) to examine whether the components of ERI differ according to socio-demographic and family-related characteristics with ERI scores ('ratio', 'effort', 'reward', and 'overcommitment') as the dependent and socio-demographic factors and family-related characteristics as the independent variables. In order to analyse the health-related impact of ERI, three separate analyses of covariance (ANCOVA) were carried out with health outcomes as the dependent continuous variables and ERI as independent variable with four categories ('none', 'slight', 'moderate', and 'marked' imbalance). With respect to the health outcome 'hypertension' logistic regression was performed due to its categorical scale level. In order to eliminate confounding effects we controlled for mothers' age, personality traits (optimism and overcommitment) and socio-demographic characteristics (income, school education, employment status, single motherhood, number of children and age of youngest child).

## Results

The age of women ranged between 17 and 60 years (mean age 39.1 ± 6.8), their youngest child ranged from 0 to 18 (mean age 9.4 ± 5.3). On average, the mothers had 1.8 children, around 40.5% of them had one child, 43.5% had two and about 16% had three and more children. Most of the women (71.6%) were married, 16.8% were single mothers. One third (32.6%) of the participants had secondary general school and about 36% got a per capita income below 926 Euro. Overall, about 78% of the mothers were employed, including mothers working up to 19 hours a week (21.8%), working half time (37.8%) and full time (17.9%). The majority of women (74.4%) reported that they are exclusively or mainly responsible for household and family work, whereas this holds only for 1% of the male counterpart. A total of 23.9% stated that their partners are equally involved in household and family work. Among women who working full time more than one in every two (55.7%) reported to bear the main burden of household and family work whereas this applied to only 4.8% of men. In total, 38.7% of full time working women stated that household and family obligations were equally shared with the partner (not displayed). Negative spillover from work to family applied to 62.7% of working women. Out of these, 41.0% perceived moderate and 21.7% marked work to family conflicts. The basic socio-demographic and family-related characteristics of the study sample are provided in Table 1.

**Table 1:** Socio-demographic and family-related characteristics of the study sample (n = 3129)

	n	%
**Mothers' age (yr)**		
17-19	4	0.1
20-29	291	9.4
30-39	1328	42.8
40-49	1339	43.2
50-60	137	4.4
Missing	30	
**School education**		
Secondary general school	1008	32.6
Intermediate secondary school	1256	40.6
Upper secondary school	830	26.8
Missing	35	
**Per capita income^1 ^(€)**		
≤ 925	959	35.5
926-1542	1212	44.9
≥ 1543	530	19.6
Missing	427	
**Number of children**		
1	1187	40.5
2	1277	43.5
>2	470	16.0
Missing	196	
**Age of youngest child (yr)**		
0-2	479	15.5
3-5	500	16.2
6-11	1008	32.6
12-15	687	22.2
16-18	420	13.6
Missing	35	
**Marital status**		
Married	2214	71.9
Married but separated	80	2.6
Single	400	13.0
Divorced	362	11.8
Widowed	24	0.8
Missing	49	
**Single motherhood**		
Yes	506	16.8
No	2511	83.2
Missing	112	
**Employment status**		
Housewife	439	14.3
Maternity leave	127	4.1
Unemployed	97	3.2
Early retirement	28	0.9
Work ≤ 19 hours/week	669	21.8
Work half time (20-37 hours/week)	1163	37.8
Work full time (≥ 38 hours/week)	550	17.9
Missing	56	
**Division of housework^2^**		
Mainly woman	1865	74.4
Woman and man alike	598	23.9
Mainly man	24	1.0
Other persons (as grandparents)	19	0.8
Missing	4	
**Spillover from work to family**		
No spillover	851	37.3
Moderate	935	41.0
Marked	494	21.7
Not employed	733	
Missing	84	
**Effort-Reward Imbalance**		
Ratio > 1	598	19.3
Ratio ≤ 1	2499	80.7
Missing	32	
**Overcommitment**		
Score > 11	737	23.8
Score ≤ 11	2358	76.2
Missing	34	

Notes: ^1 ^For calculation of per capita income information on household size and number of children were required resulting in higher frequency of missing data, ^2 ^Single mothers were excluded from calculation.

### Psychometric properties of adopted ERI questionnaire

**Figure 1:**
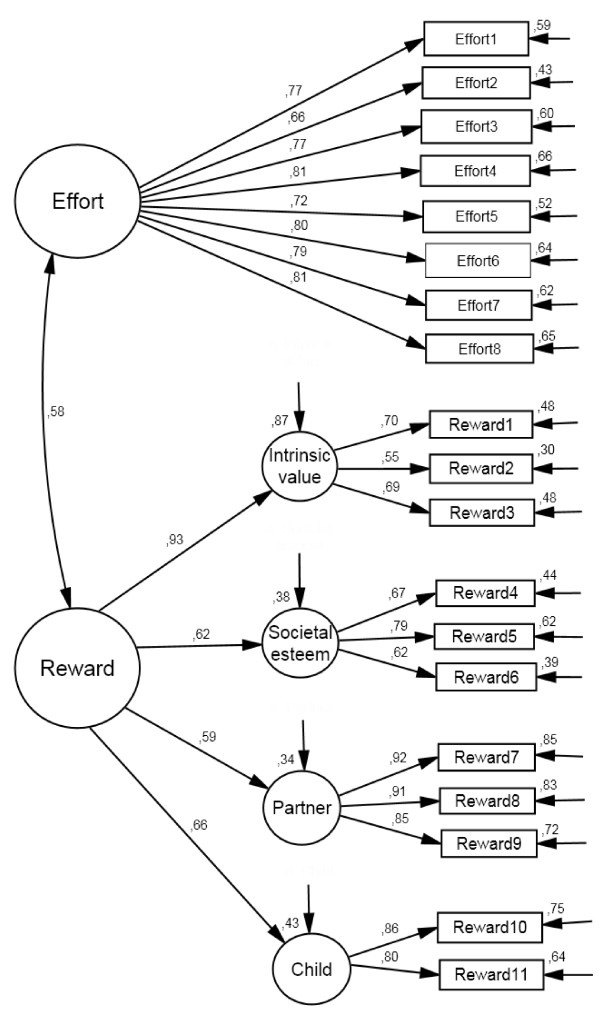
**Factorial structure of adopted ERI in household and family work with standardized regression weights (direction of the arrows to the right) and squared multiple correlations (direction of the arrows to the left and downward)**.

Confirmatory factor analysis (CFA) reproduced the theoretical structure of ERI in household and family work. As shown in Figure 1 the 'effort'-scale is based on one latent factor with eight items, whereas the scale reward is composed of four latent factors: (1) intrinsic value of household and family work (3 items), (2) societal esteem (3 items), (3) recognition from the spouse/partner (3 items), and (4) affection received by child(ren) (2 items). With the exception of 'Reward2' the model fit was appropriate with respect to standardized regression weights and squared multiple correlations. Goodness of fit (Chi²/df = 5.6) was just slightly above 5, which can be considered as satisfactory. The mean residual variances and covariances were close to the target value of zero (RMSEA = 0.04), and the model explains approximately 95 percent of observed variance and covariance (GFI = 0.96, AGFI = 0.94). Psychometric properties of the ERI scales and its components are displayed in Table 2, indicating that internal consistencies are satisfactory, ranging from 0.69 to 0.92. The same holds for the overcommitment-scale reaching high internal consistency and satisfactory fit indices in CFA (GFI = 0.99, AGFI = 0.96, RMSEA = 0.09).

**Table 2:** Statistical parameters of the ERI scales

	Effort	Reward	Intrinsic value	Societal esteem	Affection from child	Recognition from spouse	Overcom-mitment
Number of items	8	11	3	3	2	3	4

Range sumscore	8 - 40	11 - 55	3 - 15	3 - 15	2 - 10	3 - 15	4 - 16

M	20.9	22.6	5.56	7.75	3.80	6.85	9.40

SD	7.80	8.47	2.52	2.99	2.16	3.54	2.79

Cronbachs Alpha	0.92	0.84	0.69	0.73	0.82	0.82	0.81

Notes: M = Mean value, SD = Standard deviation.

### Prevalence of ERI among mothers

Table 3 displays the frequency distribution of ERI and overcommitment items. About 19.3% of mothers perceived lack of reciprocity in household and family work. With regard to 'effort' high distress due to the 'feeling as never being off duty' was reported by every third mother and is therefore the most common stressor in household and family work. All in all, the rate of mothers reporting high distress due to effort was comparatively high, ranging from 17.2% to 33.0%. Lack of reward was less frequently problematized, with lack of 'societal esteem' reaching the highest degree of approval. With respect to the personality trait 'overcommitment' 23.8% of mothers reached a sumscore ≥ 12 suggesting a psychosocial risk condition. The highest approval of almost 60% of mothers received the statements 'I easily run into time pressures' and 'already in the morning I begin to worry about family work'.

**Table 3:** Frequency distribution of ERI and Overcommitment items

		M	SD	%
	**Effort-Reward Imbalance**	0.75	0.45	19.3

	**Effort**			

Effort1	Frequently there is great time pressure	2.77	1.09	22.8

Effort2	I am frequently interrupted and disturbed	2.62	1.05	17.2

Effort3	Often I feel as never being off duty	2.77	1.34	33.0

Effort4	I would need more hours in the day to accomplish all	2.42	1.30	22.1

Effort5	My family work has become larger and larger	2.55	1.20	22.0

Effort6	I have to do a 'thousand things' all at the same time	2.90	1.14	30.7

Effort7	I'm overwhelmed by the large number of responsibilities	2.32	1.33	22.0

Effort8	I hardly get a moment's rest during the day	2.57	1.27	25.4

	**Reward: Intrinsic value**			

Reward1	I (don't) feel that family work are worth the effort	1.64	1.04	7.4

Reward2	I often question the meaning of household/family work	2.31	1.24	16.9

Reward3	The work I do for my family (don't) provide a deeper meaning to my life	1.61	0.90	4.5

	**Reward: Societal esteem**			

Reward4	Roles of housewife and mother are poorly recognized	2.71	1.19	25.5

Reward5	Nowadays, a person is regarded disapprovingly if he/she is "only" involved in household and family work	2.32	1.21	18.2

Reward6	The fact that family work is unpaid seems unjust to me	2.72	1.30	28.5

	**Reward: Recognition from the partner**			

Reward7	I (don't) obtain appropriate recognition from my partner	2.10	1.28	16.6

Reward8	Often my partner does not notice my work	2.31	1.32	21.2

Reward9	My partner (don't) often thanks me for my work at home	2.35	1.20	17.4

	**Reward: affection from the child(ren)**			

Reward10	I usually feel (not) the appreciation that I would wish for	1.95	1.13	10.5

Reward11	I (don't) receive a great deal in return from my children	1.85	1.20	12.1

	**Overcommitment**	9.40	2.80	23.8

Over1	I easily run into time pressures	2.62	0.81	58.0

Over2	Already in the morning I begin to worry about family work	2.64	0.94	58.3

Over3	I constantly think about my responsibilities at home	2.27	0.89	38.7

Over4	If I postpone something, I have trouble sleeping at night	1.88	0.84	22.3

Notes: M = Mean value, SD = Standard deviation, %= Percentage of mothers reporting ERI (ratio > 1), high overcommitment (categories 3 and 4 of response format, sumscore ≥ 12) and high distress due to high effort and low reward (categories 4 and 5 of response format).

### Associations between ERI components and socio-demographic characteristics

Table 4 shows the relationships between ERI and socio-demographic factors. According to mothers' age, effort as well as reward scores decreased when women are getting older. Consequently, effort-reward ratio scores remained largely stable over different age groups, while overcommitment in terms of 'inability to withdraw from work obligations' decreased with mothers' age. With respect to school education the findings suggest that mothers with lower educational degrees perceived less reward in household and family work while higher educated mothers received the highest reward, but also the highest effort scores. Mothers with intermediate secondary school degree had the lowest ERI scores. A pronounced social gradient in the ratio score was found with respect to income: low-income mothers perceived less reward and exhibited also higher effort scores as well as overcommitment scores compared with more affluent mothers. Effort scores increased when mothers bring up more children, while reward scores moderately decreased with number of children. Ratio scores were highest when the youngest child is between three and five years and tended to decrease when children are getting older. The effort-reward imbalance scores were somewhat lower among housewives due to lower effort scores as compared with mothers working half and full-time. Unemployed mothers more often lacked of reward in particular compared with mothers working full-time. Also single motherhood was making women prone to lack of reciprocity. Compared with partnered mothers they perceived less reward and they also had higher levels of effort and overcommitment. Perceived inequality in the division of domestic work had a considerable effect on all ERI components. Mothers who reported to bear the main burden of household and family work reached in particular significant lower reward scores compared to women whose partners are equally involved in household and family work. Also negative work to family spill over has a superior effect on effort-reward imbalance score. This holds for all components of the model but especially for the effort scale.

**Table 4:** Associations between ERI and social and family-related characteristics

	Ratio		Effort		Reward		Overcommitment	
**Mothers' age (yr)**	**M**	**SD**	**M**	**SD**	**M**	**SD**	**M**	**SD**
< 29	0.73	0.39	21.06	7.56	43.15	7.93	9.57	2.56
30-39	0.77	0.44	21.60	7.41	42.26	8.19	9.71	2.80
40-49	0.74	0.46	20.32	8.09	41.95	8.34	9.08	2.80
50-60	0.72	0.54	18.77	9.22	41.83	9.43	8.83	2.90
	*F = 1.92*	*p = .383*	*F = 9.41*	*p <.001*	*F = 1.84*	*p = .138*	*F = 13.55*	*p < .001*

**School education**								
Secondary general	0.77	0.49	20.69	8.21	41.01	8.60	9.44	2.94
Intermediate secondary	0.73	0.42	20.64	7.76	42.52	8.14	9.28	2.75
Upper secondary	0.75	0.45	21.53	7.47	43.09	8.28	9.50	2.69
	*F = 2.71*	*p = .067*	*F = 3.73*	*p = .021*	*F = 16.79*	*p < .001*	*F = 1.65*	*p = .192*

**Per capita income**								
≤ 925	0.79	0.47	21.23	7.98	40.99	8.68	9.60	2.86
926-1542	0.74	0.42	20.94	7.57	42.22	8.00	9.36	2.71
≥ 1543	0.69	0.41	20.56	7.99	44.24	7.32	9.16	2.83
	*F = 9.88*	*p < .001*	*F = 1.33*	*p = .264*	*F = 28.86*	*p < .001*	*F = 4.68*	*p = .009*

**Number of children**								
1	0.71	0.44	20.08	7.88	42.86	8.28	9.20	2.82
2	0.77	0.46	21.30	7.70	41.93	8.27	9.50	2.72
*>2*	0.80	0.43	21.80	7.85	41.27	8.48	9.59	2.99
	*F = 8.58*	*p < .001*	*F = 11.44*	*p < .001*	*F = 7.07*	*p < .001*	*F = 4.75*	*p = .009*

**Age of youngest child (yr)**								
0-2	0.73	0.37	21.70	6.75	43.54	7.36	9.52	2.62
3-5	0.80	0.45	22.47	7.60	42.43	7.90	9.98	2.76
6-11	0.76	0.41	21.42	7.66	42.12	8.04	9.49	2.81
12-15	0.75	0.52	19.83	8.45	41.28	8.98	9.10	2.90
16-18	0.68	0.46	18.36	7.83	42.13	8.71	8.79	2.73
	*F = 4.48*	*p < .001*	*F = 22.87*	*p < .001*	*F = 5.54*	*p < .001*	*F = 13.32*	*p < .001*

**Single motherhood^1^**								
*Yes*	0.84	0.53	21.81	8.47	29.50	6.67	9.77	2.95
*No*	0.73	0.44	20.73	7.68	31.28	5.72	9.32	2.77
	*F = 24.87*	*p < .001*	*F = 8.31*	*p=.004*	*F = 38.70*	*p < .001*	*F = 11.37*	*p = .001*

**Employment status**								
Housewife	0.71	0.44	19.74	7.54	42.10	7.91	9.12	2.78
Unemployed	0.76	0.41	20.59	7.68	40.11	8.36	9.73	2.87
Work ≤ 19 hours/week	0.76	0.45	20.56	7.86	41.18	8.56	9.37	2.84
Work 20-37 hours/week	0.76	0.43	21.38	7.72	42.34	8.17	9.43	2.78
Work ≥ 38 hours/week	0.72	0.45	20.92	8.21	43.88	7.98	9.41	2.83
	*F = 2.04*	*p = .058*	*F = 3.09*	*p = .005*	*F = 7.58*	*p < .001*	*F = 1.01*	*p = .416*

**Division of housework^2^**								
Mainly woman	0.79	0.44	21.60	7.64	41.25	8.06	9.56	2.77
Woman and man alike	0.57	0.31	18.13	7.21	46.39	6.50	8.63	2.61
	*F = 133.73*	*p < .001*	*F = 99.59*	*p < .001*	*F = 208.01*	*p < .001*	*F = 54.10*	*p < .001*

**Spillover work to family^3^**								
No spillover	0.64	0.40	18.36	7.41	43.51	7.98	8.74	2.74
Moderate	0.76	0.36	21.60	6.77	42.91	7.58	9.55	2.58
Marked	1.13	0.53	28.00	6.43	37.70	8.92	11.32	2.49
	*F = 272.85*	*p < .001*	*F = 362.55*	*p < .001*	*F = 100.41*	*p < .001*	*F = 186.70*	*p < .001*

Notes: To give a better understanding different to Table 2 and 3 reward items are coded in such a way that low values indicate low reward, while high values mean high reward. 1 The subscale 'recognition from the spouse' is not applicable to single mothers. Thus here, the scale 'reward' only comprises the remaining three subscales 'affection from the child', 'societal esteem' and 'intrinsic value'. The comparative analysis with partnered mothers has also been carried out with the reduced reward scale. ^2^Categories 'mainly man' and 'other persons' have been neglected due to small number of valid cases (see Table 1), single mothers were excluded from calculation. ^3^Women not working were assigned to the group 'no spillover'. M = Mean Value, SD = Standard deviation.

### Impact of ERI on mothers' health

**Figure 2:**
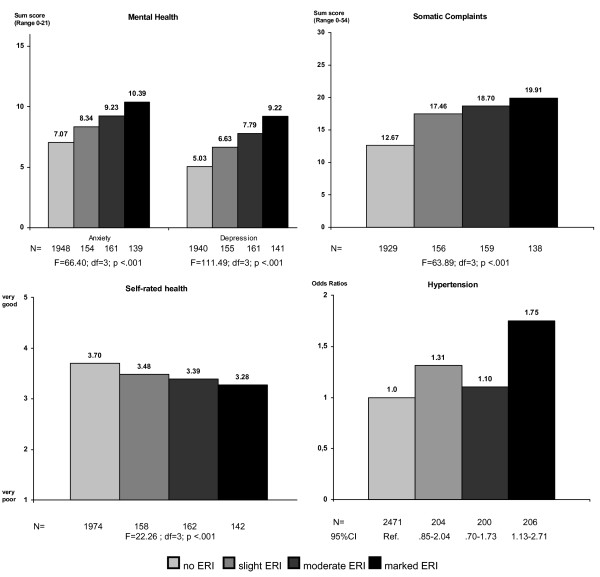
**Effects of ERI on mother's health outcomes**. ANCOVA and logistic regression statistics adjusted for age, personality traits and socio-demographic characteristics. Notes: Number of missing values: anxiety:n = 50 (1.6%), depression n = 52 (1.7%), somatic complaints n = 96 (3.1%), self-rated health n = 15 (0.5%) and hypertension n = 16 (0.5%)

Mothers experiencing an imbalance between efforts and rewards in household and family work reported poorer health compared to those who did not perceived such kind of mismatch. As Figure 2 illustrates, there is a linear association between ERI and most of the health outcomes in a way that health impairments continuously increased with ERI. The strongest association was found for mental health: mothers reporting no lack of reciprocity had significantly lower anxiety and depression levels as compared to those reporting high levels of imbalance. The same but less pronounced also holds true for somatic complaints and self-rated health. With respect to blood pressure solely a marked imbalance was associated with a significantly higher rate of hypertension.

## Discussion

The application of effort-reward imbalance (ERI) to household and family work provides an access to information on work at home similar to what was reported for paid work. Although there are crucial differences between paid and unpaid work, we postulate that nonreciprocal exchange in household and family work may lead to similar emotional distress caused by the fact of being treated unfairly in the role as a wife and mother. In a recent study, a questionnaire for assessing effort-reward imbalance in household and family work was developed and tested on clinical data of mothers with burnout. Analogous to the original version, the newly developed questionnaire contains an extrinsic (dysbalance of effort and reward) as well as an intrinsic component (overcommitment). For assessing overcommitment only moderate linguistic changes from the original questionnaire were needed. In this study the factorial validity of this questionnaire was evaluated using data of a population sample of mothers with minor children. The findings confirmed the factorial structure of the theoretical ERI model. The subscale 'reward' is devided into four dimensions: 'intrinsic value of household and family work', 'societal esteem', 'recognition from the partner', and 'affection from the child(ren)'. Though overcommitment is part of the ERI-model the theoretical position of this component is not completely clear so far. Originally, overcommitment was conceptualized as the intrinsic part of effort. In this view, highly overcommitted employees tend to invest too many efforts. Therefore, the amount of effort invested is dependent upon extrinsic demands as well as intrinsic efforts. Meanwhile overcommitment is seen as an independent concept which influences the perception of both, high efforts and low rewards [3]. According to this current approach overcommitment mediates the relationship between the effort-reward ratio and health outcomes. Therefore, we kept overcommitment out of confirmatory factor analysis and controlled for this personality characteristic when analysing the impact of the ratio on health. However, in a recent publication overcommitment is regarded as an integral part of ERI [20]. Thus, clarification about theoretical conceptualization of overcommitment is needed in order to realize a more coherent application of the ERI model.

Irrespectively of how overcommitment was conceptualized, our findings revealed that a significant proportion of mothers showed high rates of overcommitment in terms of 'inability withdraw from household and family work obligations'. We also found high rates of effort, expressed particularly by the feeling 'as never being off duty' and 'having to do a thousand things all at the same time'. Lack of reward was less relevant, but statements concerning less 'societal esteem' received considerable approval. This supports findings from earlier studies suggesting that the value of being a housewife and mother has declined over the last decades [8].

Our analyses have shown that ERI differs according to socio-demographic factors and family characteristics. As expected, ERI ratio scores increased with the number of children and decreased when children are getting older. Beside child-related risk factors also socioeconomic status seems to affect the mismatch between requested efforts and given rewards. Our findings suggest that in particular low income is associated with higher effort as well as with noticeably lower reward, resulting in higher ERI scores. A high mismatch was also found for single mothers. This indicates that ERI in household and family work may particularly capture the stress experience of socially disadvantaged mothers.

Through increased participation in the labour force, combining work and family demands are an increasingly common source of conflict for women [21]. Negative work to family spillover describes the fact that employment obligations adversely affect family obligations. Several studies have pointed out that negative work to family spillover hamper marital satisfaction and functioning [22]. Our analyses revealed that employment status per se was of minor relevance for women's perception of ERI. However, high levels of negative work to family spillover turned out to have a superior effect on ERI scores, in particular with respect to the 'effort' component. For future research it would be interesting to assess ERI at work and at home simultaneously. This approach may provide a more comprehensive picture of women's total daily stress perception and may give insights into the interplay between stress experiences in both domains.

In line with Bebbington [23] our findings suggest that women still perform the majority of the household and family work even when they are working fulltime. Perceived inequality in the division of household and family work was of major importance for high ERI-scores in women. Interestingly, different to the effect of negative spillover, men's lack of engagement did not primarily affect the perception of effort, but of reward. Thus, inequality in divison of domestic work seems to go along with women's perception of undervaluing their work. As Siegrist [24] pointed out, recurrent experience of reward deficiency in a core social role impairs successful self-regulation and in the long run increases illness susceptibility. Analysis of the health-related impact of ERI confirmed that mothers experiencing an imbalance between efforts and rewards at home are at higher risk of ill health. With the exception of hypertension we found a linear association between ERI and health outcomes such that health impairments continuously increased with ERI. This result provides first evidence that ERI in household and family work defines a state of emotional distress with negative health consequences, analogous to ERI at work. Given that disadvantaged mothers are at higher risk of ERI in household and family work, this concept may provide a complementary framework for explaining the social gradient in women's health. However, further analyses are needed in order to specify the role of ERI for explaining health inequalities among mothers.

Finally, some crucial limitations of the study need to be addressed. As our study is based exclusively on subjective data, the association between ERI and health outcomes should be interpreted cautiously, as there is some evidence that individual differences in personality traits may affect the reporting of stressors and health problems alike [25]. Additionally, with our cross-sectional study design we cannot draw conclusions about causality, but we assume in line with literature [1-3] that ERI acts on health rather than the other way around. In order to minimize sources of error caused by self-reported measures, we adjusted for pessi-mism and overcommitment as personality traits that may influence mother's responses. However, further investigations with repeated measures over time as well as more objective measures of mental health are needed in order to further validate the findings. Finally, it has to be mentioned that the study was performed by means of an access panel sample survey. Access panel designs are composed of respondents giving their general consent to participate in surveys. Hence, besides usual response bias due to lack of controllability of target persons also selection bias due to the preselection of participants may reduce the representativeness of our study. On the other hand, using access panel data allows control over sample coverage with respect to socio-demographic and household characteristics. In addition, this panel facilitates access to target groups that are otherwise difficult to reach. In particular this applies to mothers with minor children. In light of the declining willingness to participate in surveys [26] access panel surveys may become more important in the future. This underlines the need for more basic research in order to assess the representativeness of such a sample type.

## Conclusions

In sum, it can be stated that the newly developed questionnaire was shown to have high validity according to its factorial structure, and promising results for extending the ERI model to household and family work were obtained. Further analyses are necessary in order to clarify whether ERI may provide an appropriate theoretical approach to depict health-related stress experiences in household and family work.

## Competing interests

The authors declare that they have no competing interests.

## Authors' contributions

SS conceived of the study design and carried out the study. She drafted the major part of manuscript and performed the statistical analysis. RP participated in the design of the study and has been involved in revising the manuscript critically for important intellectual content. He also participated in statistical analysis and interpretation of data. SG has made substantial contributions to conception and design of the study and helped to draft the manuscript. All authors read and approved the final manuscript.

## Pre-publication history

The pre-publication history for this paper can be accessed here:

http://www.biomedcentral.com/1471-2458/12/12/prepub

## Supplementary Material

Additional file 1 **Questionnaires**. Click here for file
