# Supplementary material for: Applying the effort-reward imbalance model to household and family work: a population-based study of German mothers
Source: BMC Public Health. 2012 Jan 6;12:12. doi: 10.1186/1471-2458-12-12 (PMC3293094; doi:10.1186/1471-2458-12-12)
Supplement: Additional file 1 — Questionnaires. [file 1471-2458-12-12-S1.DOC]

**Appendix:** **Questionnaires**

*Overcommitment*

Over1 I easily run into time pressures in my household and family work.

Over2 From the moment I wake up in the morning, I often begin to worry about household and family work that needs to be completed.

Over3 I constantly think about my responsibilities at home, and I’m still preoccupied with them in the evening.

Over4 If I postpone something that I really should have finished today, I have trouble sleeping at night.

*Effort-Reward Imbalance (ERI) in household and family work*

*Effort*

Effort1 Frequently there is great time pressure due to the many tasks in household and for my family.

Effort2 I am frequently interrupted and disturbed in my activities in the household and for my family.

Effort3 Often I feel as never being off duty.

Effort4 I would need more hours in the day in order to accomplish all my household and family work.

Effort5 Over the last years, my household and family work has become larger and larger.

Effort6 In household and family work, I often have the feeling of having to accomplish “a thousand things” all at the same time.

Effort7 I often feel overwhelmed by the large number of household and family responsibilities.

Effort8 I hardly get a moment’s rest during the day because of the many demands placed on me by the household and my family.

*Reward*

*Component intrinsic value*

Reward1 I feel that overall, house and family work are worth the effort.

Reward2 I often question the meaning of household and family work, since I have to start all over again every day.

Reward3 The work I do for my family provides a deeper meaning to my life.

*Component societal esteem*

Reward4 In my interactions with other people, I often have the experience that the roles of housewife and mother are poorly recognized and appreciated.

Reward5 Nowadays, a person is regarded disapprovingly if he/she is “only” involved in household and family work.

Reward6 The fact that household and family work are unpaid seems unjust to me.

*Component partner*

Reward7 I usually obtain an appropriate level of recognition and appreciation from my partner for my work at home.

Reward8 Often my partner does not notice my work in the household and for the family.

Reward9 My partner often thanks me for my work at home.

*Component child*

Reward10 From my child/children I usually feel the appreciation and affection that I would wish for.

Reward11 I receive a great deal in return from my children/child for my efforts at home.
